# K(MoO_2_)_4_O_3_(AsO_4_)

**DOI:** 10.1107/S1600536813013664

**Published:** 2013-05-25

**Authors:** Raja Jouini, Mohamed Faouzi Zid, Ahmed Driss

**Affiliations:** a Laboratoire de Matériaux et Cristallochimie, Faculté des Sciences de Tunis, Université de Tunis ElManar, 2092 ElManar II Tunis, Tunisia

## Abstract

A new compound with a non-centrosymmetric structure, potassium tetra­kis­[dioxomolybdenum(IV)] arsenate trioxide, K(MoO_2_)_4_O_3_(AsO_4_), has been synthesized by a solid-state reaction. The [(MoO_2_)_4_O_3_(AsO_4_)]^+^ three-dimensional framework consists of single arsenate AsO_4_ tetra­hedra, MoO_6_ octa­hedra, MoO_5_ bipyramids and bi­octa­hedral units of edge-sharing Mo_2_O_10_ octa­hedra. The [Mo_2_O_8_]_∞_ octa­hedral chains running along the *a-*axis direction are connected through their corners to the AsO_4_ tetra­hedra, MoO_6_ octa­hedra and MoO_5_ bipyramids, so as to form large tunnels propagating along the *a* axis in which the K^+^ cations are located. This structure is compared with compounds containing *M*_2_O_10_ (*M* = Mo, V, Fe) dimers and with those containing *M*_2_O_8_ (*M* = V) chains.

## Related literature

For background to the physico-chemical properties of related compounds, see: Piffard *et al.* (1985 ▶); Centi *et al.* (1988 ▶); Stucky *et al.* (1989 ▶); Northrup *et al.* (1994 ▶); Ouerfelli *et al.* (2007 ▶). For details of structurally related compounds, see: Amoros & LeBail (1992 ▶), Boudin *et al.* (1994 ▶); Guesdon *et al.* (1994 ▶, 1995 ▶); Borel *et al.* (1994 ▶, 2000 ▶); LeBail *et al.* (1995 ▶); Berrah *et al.* (1999 ▶); Hajji *et al.* (2009 ▶). For the preparation, see: Jouini *et al.* (2012 ▶). For bond-valence sums, see: Brown & Altermatt (1985 ▶).

## Experimental

### 

#### Crystal data

K(MoO_2_)_4_O_3_(AsO_4_)
*M* *_r_* = 737.78
Orthorhombic, 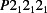

*a* = 8.0442 (8) Å
*b* = 11.9167 (9) Å
*c* = 12.6799 (10) Å
*V* = 1215.50 (18) Å^3^
*Z* = 4
Mo *K*α radiation
μ = 7.16 mm^−1^
*T* = 298 K
0.42 × 0.33 × 0.22 mm

#### Data collection

Enraf–Nonius CAD-4 diffractometer
Absorption correction: ψ scan (North *et al.*, 1968 ▶) *T*_min_ = 0.077, *T*_max_ = 0.212
8046 measured reflections
2647 independent reflections
2611 reflections with *I* > 2σ(*I*)
*R*_int_ = 0.030
2 standard reflections every 120 min intensity decay: 1.2%

#### Refinement

*R*[*F* ^2^ > 2σ(*F* ^2^)] = 0.018
*wR*(*F* ^2^) = 0.045
*S* = 1.10
2647 reflections
191 parameters
Δρ_max_ = 0.52 e Å^−3^
Δρ_min_ = −0.69 e Å^−3^
Absolute structure: Flack (1983 ▶), 2399 Friedel pairs
Flack parameter: 0.089 (10)

### 

Data collection: *CAD-4 EXPRESS* (Duisenberg, 1992 ▶; Macíček & Yordanov, 1992 ▶); cell refinement: *CAD-4 EXPRESS*); data reduction: *XCAD4* (Harms & Wocadlo, 1995 ▶); program(s) used to solve structure: *SHELXS97* (Sheldrick, 2008 ▶); program(s) used to refine structure: *SHELXL97* (Sheldrick, 2008 ▶); molecular graphics: *DIAMOND* (Brandenburg, 1998 ▶); software used to prepare material for publication: *WinGX* (Farrugia, 2012 ▶).

## Supplementary Material

Crystal structure: contains datablock(s) I, global. DOI: 10.1107/S1600536813013664/vn2070sup1.cif

Structure factors: contains datablock(s) I. DOI: 10.1107/S1600536813013664/vn2070Isup2.hkl

Additional supplementary materials:  crystallographic information; 3D view; checkCIF report

## Figures and Tables

**Fig. 1.**
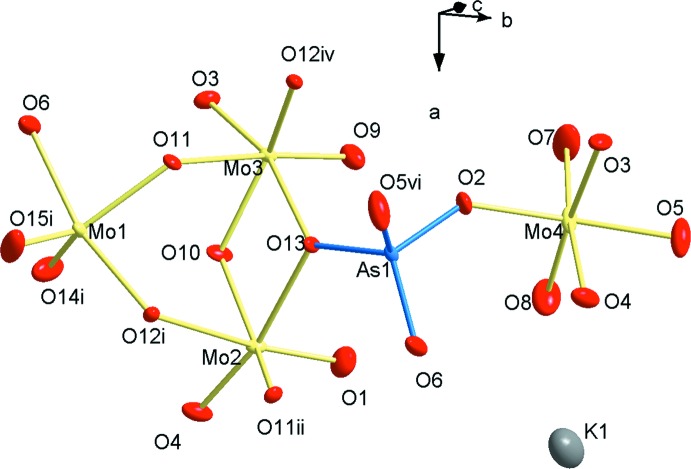
Unité asymétrique dans K(MoO2)4O3(AsO4). Les ellipsoïdes ont été définis avec 50% de probabilités. [codes de symétrie: (i) 1 + x, y, z; (ii) 3/2 - x, 1 - y, 1/2 + z; (iii) 1 - x, 1/2 + y, 3/2 - z; (iv) 1/2 - x, 1 - y, 1/2 + z; (v) -1 + x, y, z; (vi) 1 - x, -1/2 + y, 3/2 - z].

**Fig. 2.**
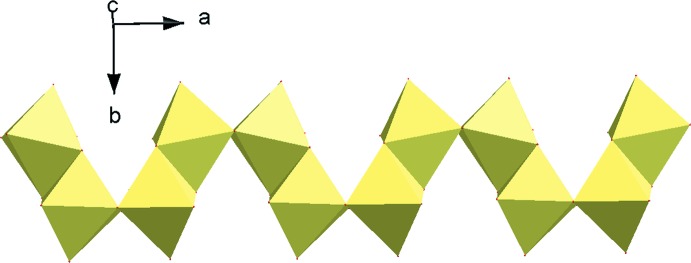
Projection de chaînes ondulées de type Mo2O8 selon la direction [100].

**Fig. 3.**
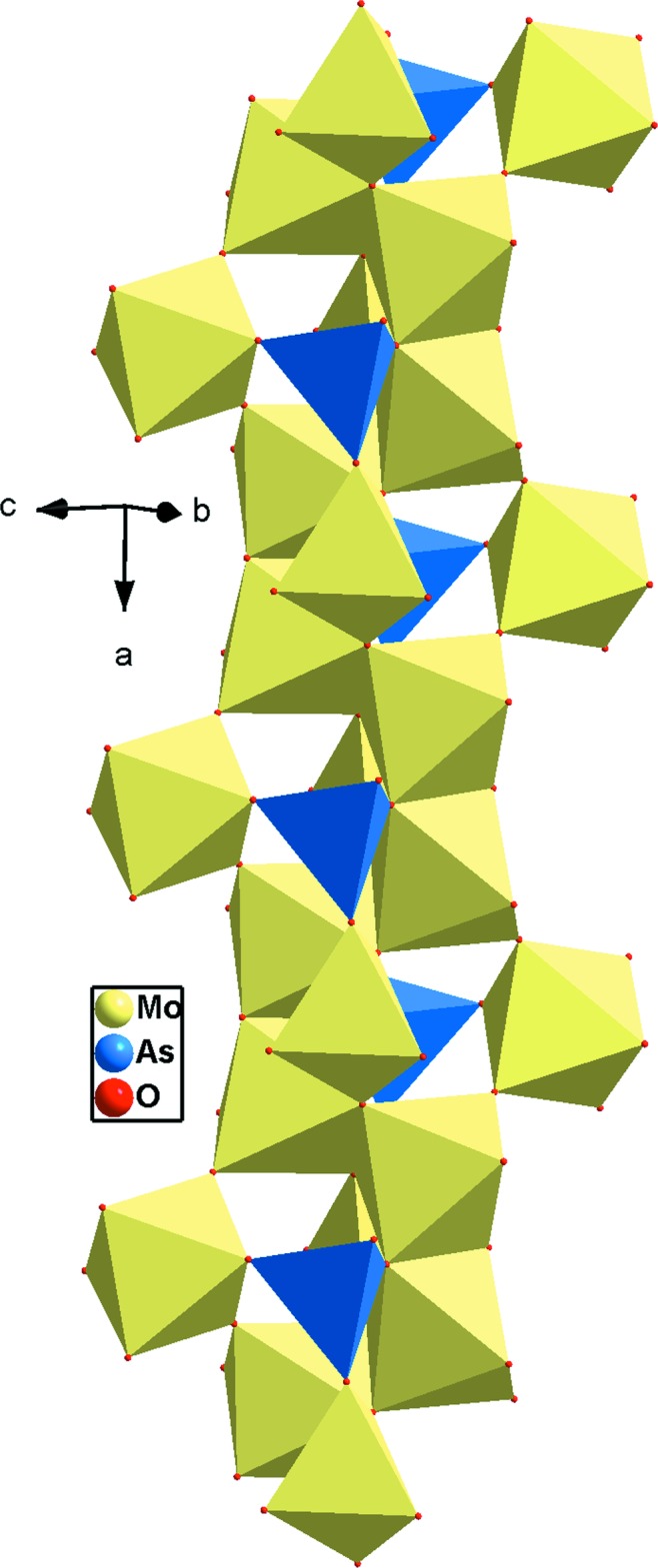
Projection des rubans de type Mo8AsO34 selon la direction [100].

**Fig. 4.**
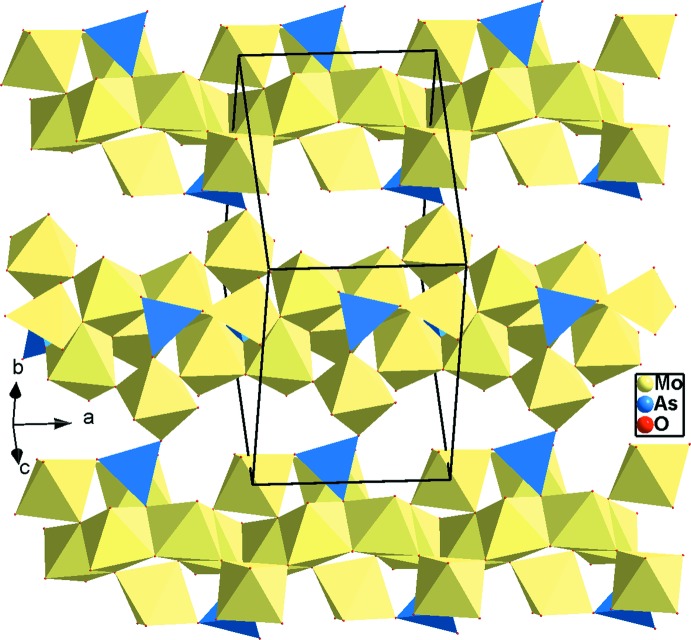
Représentation de la cohésion entre les rubans.

**Fig. 5.**
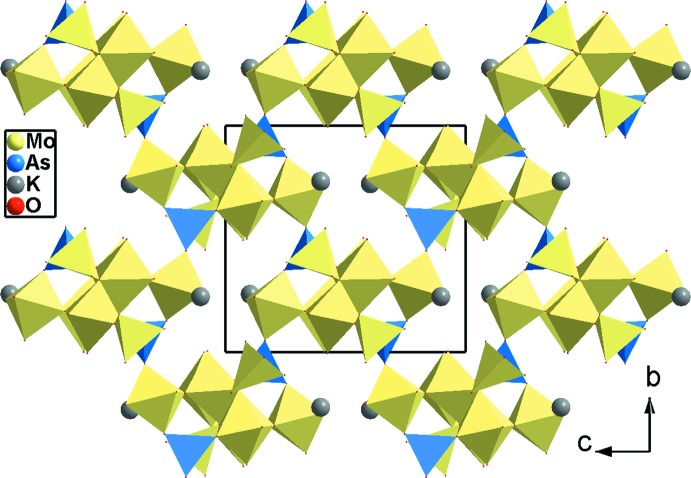
Projection de la structure de K(MoO2)4O3(AsO4) selon a, mettant en évidence les canaux où logent les atomes de potassium.

## supplementary crystallographic information

### Comment

Diverses études sur des composés à charpentes covalentes formées d'octaèdres et de tétraèdres ont débuté il y a plusieurs années. Les applications espérées des nouveaux oxydes élaborés sont en catalyse hétérogène comme produits d'intercalation (Centi *et al.*, 1988) et comme échangeurs cationiques (Piffard *et al.*, 1985) et conducteurs ioniques (Ouerfelli *et al.*, 2007). Ces matériaux peuvent être aussi dotés de propriétés d'optique non-linéaire pour les composés non-centrosymétriques (Northrup *et al.*, 1994).

À titre de contribution à l'étude de ces types de matériaux, nous avons poursuivi l'exploration des systèmes A—Mo—As—O (A= cation monovalent) dans lesquels nous avons précédemment caractérisé la phase suivante: Na_2_MoO_2_As_2_O_7_ (Jouini *et al.*, 2012) dans le but de trouver des nouveaux arséniates qui manifestent des caractéristiques structurales favorables à une bonne conduction ionique. Une nouvelle phase non-centrosymétrique de formulation K(MoO_2_)_4_O_3_(AsO_4_) a été synthétisée par réaction à l'état solide. L'unité asymétrique dans la structure est constituée de deux octaèdres MoO_6_ reliés par partage d'une arête formant ainsi le bioctaèdre Mo_2_O_10_. Ce dernier met en commun deux de ses sommets avec une bipyramide Mo1O_5_ et un autre sommet avec un tétraèdre AsO_4_ relié lui-même à un octaèdre Mo4O_6_ par partage d'un sommet (Fig. 1). La charpente anionique peut être décrite à partir des chaînes ondulées infinies (Mo_2_O_8_)∞ formées par les dimères Mo_2_O_10_ reliés par partage d'arêtes selon la direction [100] (Fig. 2). Au sein de ces chaînes, chaque dimère partage deux de ses sommets avec deux octaèdres Mo4O_6_, quatre sommets avec trois bipyramides Mo1O_5_ et un sommet avec un tétraèdre AsO_4_ laissant libre trois sommets. Cette association conduit à des rubans de type Mo_8_AsO_34_ disposés selon la direction [100] (Fig. 3). La cohésion entre ces derniers est assurée par formation de ponts mixtes Mo—O—As (Fig. 4). L'association de ces rubans conduit à une structure tridimensionnelle possédant de larges canaux. Les atomes de potassium sont situés dans les canaux mais excentrés (Fig. 5). Les valeurs des charges des ions (BVS) dans la structure ont été calculées moyennant la formule empirique de Brown (Brown & Altermatt, 1985). Le résultat final est: Mo1(6,080), Mo2(6,143), Mo3(6,086), Mo4(6,153), As1(5,292) et K1(1,222) confirme les degrés d'oxydation des différents ions dans la phase étudiée.

La comparaison de la structure de K(MoO_2_)_4_O_3_(AsO_4_) avec celles trouvées dans la littérature et renfermant le même groupement Mo_2_O_10_ montre que ces dimères sont rencontrés dans les monophosphates de formulation *A*Mo_3_P_2_O_14_ (*A*=Na (Borel *et al.*, 1994); *A*=K (Guesdon *et al.*, 1994)), dans le composé Cs_3_Mo_8_O_11_(PO_4_)_8_ (Borel *et al.*, 2000), dans le diphosphate β-K_2_Mo_2_O_4_P_2_O_7_ (Guesdon *et al.*, 1995) ainsi que dans l'arséniate Li_3_AlMo_2_As_2_O_14_ (Hajji *et al.*, 2009). Les monophosphates de formulation *A*Mo_3_P_2_O_14_ présentent des structures bidimensionnelles où les groupements Mo_2_O_10_ partagent leurs sommets avec les polyèdres PO_4_ et MoO_6_. Dans la charpente tridimensionnelle de Cs_3_Mo_8_O_11_(PO_4_)_8_, chaque unité Mo_2_O_10_ partage sept sommets avec les tétraèdres PO_4_, alors que dans le diphosphate β-K_2_Mo_2_O_4_P_2_O_7_, chaque groupement Mo_2_O_10_ met en commun six sommets avec cinq groupements P_2_O_7_. Le matériau Li_3_AlMo_2_As_2_O_14_ est caractérisé par une structure très ouverte liée au fait que chaque groupement Mo_2_O_10_ ne partage ses sommets qu'avec seulement deux tétraèdres AsO_4_ et deux octaèdres AlO_6_. Dans le composé FeVMoO_7_ (LeBail *et al.*, 1995), les dimères *M*_2_O_10_ sont observés sous forme de Fe_2_O_10_ où ce dernier partage ses dix sommets avec respectivement quatre tétraèdres MoO_4_ et six tétraèdres VO_4_ conduisant à une charpente bidimensionnelle. Certains phosphates de vanadium sont caractérisés par la présence des groupements V_2_O_10_ comme Cd_3_V_4_(PO_4_)_6_ (Boudin *et al.*, 1994) dans lequel les dimères V_2_O_10_ sont connectés directement aux groupements PO_4_. Une différence est observée dans le comportement des dimères dans ces matériaux et ceux existant dans notre composé. En effet, chaque dimère dans notre matériau partage ses sommets avec trois polyèdres de nature différentes: deux de ses sommets sont liés à deux octaèdres MoO_6_, quatre sommets avec trois bipyramids MoO_5_ et un sommet avec un tétraèdre AsO_4_ laissant ainsi libre trois sommets et conduisant à une structure tridimensionnelle. Notre structure est construite aussi à partir de chaînes ondulées Mo_2_O_8_. Ce type de chaînes *M*_2_O_8_ est observé sous forme de V_2_O_8_ dans d'autres composés rencontrés dans la bibilographie notamment: KV_2_PO_8_ (Berrah *et al.*, 1999) et l'hydrogénophosphate d'ammonium α-NH_4_VO_2_PO_3_OH (Amoros & LeBail, 1992), dans lesquels les pyramides VO_5_ sont connectés les uns aux autres au moyen de sommets. Contrairement à notre composé où les octaèdres MoO_6_ se lient par partage d'arêtes formant des chaînes ondulées. Le composé K(MoO_2_)_4_O_3_(AsO_4_) appartenant à une classe non-centrosymétrique (groupe d'espace: *P*2_1_2_1_2_1_) pourrait présenter des propriétés d'optique non-linéaires (Stucky *et al.*, 1989).

### Experimental

Les cristaux relatifs à la phase K(MoO_2_)_4_O_3_(AsO_4_) ont été obtenus à partir des réactifs solides K_2_CO_3_ (Fluka, 69858), (NH_4_)_2_Mo_4_O_13_ (Fluka, 69858) e t NH_4_H_2_AsO_4_ (préparé au laboratoire, ASTM 01–775) pris dans les proportions K:Mo:As=1:4:1. Le mélange a été finement broyé et préchauffé à l'air à 573 K pendant une nuit. Après refroidissement et broyage, la préparation est portée, proche de la fusion à 823 K pour favoriser la germination et la croissance des cristaux, pendant une semaine. Le résidu final est refroidi lentement (5°/demi journée, à 773 K) puis rapide (50°/h) jusqu'à la température ambiante. Par lavage à l'eau chaude des cristaux de couleur jaunâtre de qualité et de taille suffisante ont été séparés pour analyse par DRX.

### Refinement

Les densités d'électrons maximum et minimum restants dans la Fourier-différence sont acceptables et sont situées respectivements à 0.80 Å de O9 et à 0.56 Å de K1.

### Crystal data

**Table:**

K(MoO_2_)_4_O_3_(AsO_4_)	*F*(000) = 1360
*M**_r_* = 737.78	*D*_x_ = 4.032 Mg m^−^^3^
Orthorhombic, *P*2_1_2_1_2_1_	Mo *K*α radiation, λ = 0.71073 Å
Hall symbol: P 2ac 2ab	Cell parameters from 25 reflections
*a* = 8.0442 (8) Å	θ = 10–15°
*b* = 11.9167 (9) Å	µ = 7.16 mm^−^^1^
*c* = 12.6799 (10) Å	*T* = 298 K
*V* = 1215.50 (18) Å^3^	Prism, yellow
*Z* = 4	0.42 × 0.33 × 0.22 mm

### Data collection

**Table:**

Enraf–Nonius CAD-4 diffractometer	2611 reflections with *I* > 2σ(*I*)
Radiation source: fine-focus sealed tube	*R*_int_ = 0.030
Graphite monochromator	θ_max_ = 27.0°, θ_min_ = 2.4°
ω/2θ scans	*h* = −10→4
Absorption correction: ψ scan (North *et al.*, 1968)	*k* = −15→15
*T*_min_ = 0.077, *T*_max_ = 0.212	*l* = −16→16
8046 measured reflections	2 standard reflections every 120 min
2647 independent reflections	intensity decay: 1.2%

### Refinement

**Table:**

Refinement on *F*^2^	Secondary atom site location: difference Fourier map
Least-squares matrix: full	*w* = 1/[σ^2^(*F*_o_^2^) + (0.0195*P*)^2^ + 2.5252*P*] where *P* = (*F*_o_^2^ + 2*F*_c_^2^)/3
*R*[*F*^2^ > 2σ(*F*^2^)] = 0.018	(Δ/σ)_max_ = 0.001
*wR*(*F*^2^) = 0.045	Δρ_max_ = 0.52 e Å^−^^3^
*S* = 1.10	Δρ_min_ = −0.69 e Å^−^^3^
2647 reflections	Extinction correction: *SHELXL97* (Sheldrick, 2008), Fc^*^=kFc[1+0.001xFc^2^λ^3^/sin(2θ)]^-1/4^
191 parameters	Extinction coefficient: 0.00488 (17)
0 restraints	Absolute structure: Flack (1983), 2399 Friedel pairs
Primary atom site location: structure-invariant direct methods	Flack parameter: 0.089 (10)

### Special details

**Table:**

Geometry. All e.s.d.'s (except the e.s.d. in the dihedral angle between two l.s. planes) are estimated using the full covariance matrix. The cell e.s.d.'s are taken into account individually in the estimation of e.s.d.'s in distances, angles and torsion angles; correlations between e.s.d.'s in cell parameters are only used when they are defined by crystal symmetry. An approximate (isotropic) treatment of cell e.s.d.'s is used for estimating e.s.d.'s involving l.s. planes.
Refinement. Refinement of *F*^2^ against ALL reflections. The weighted *R*-factor *wR* and goodness of fit *S* are based on *F*^2^, conventional *R*-factors *R* are based on *F*, with *F* set to zero for negative *F*^2^. The threshold expression of *F*^2^ > σ(*F*^2^) is used only for calculating *R*-factors(gt) *etc*. and is not relevant to the choice of reflections for refinement. *R*-factors based on *F*^2^ are statistically about twice as large as those based on *F*, and *R*- factors based on ALL data will be even larger.

### Fractional atomic coordinates and isotropic or equivalent isotropic displacement parameters (Å^2^)

**Table:**

	*x*	*y*	*z*	*U*_iso_*/*U*_eq_	
Mo1	0.99047 (5)	0.41688 (3)	0.61974 (3)	0.01100 (9)	
Mo2	0.78403 (5)	0.64073 (3)	0.90494 (3)	0.00936 (9)	
Mo3	0.19469 (5)	0.63578 (3)	0.92886 (3)	0.00829 (9)	
Mo4	0.46142 (5)	0.71544 (3)	0.68083 (3)	0.01289 (10)	
As1	0.56223 (6)	0.43948 (3)	0.65276 (3)	0.00879 (10)	
K1	0.9351 (2)	0.75546 (14)	0.60338 (12)	0.0474 (4)	
O1	0.7659 (5)	0.5009 (3)	0.9106 (3)	0.0236 (8)	
O2	0.4420 (4)	0.5521 (2)	0.6643 (3)	0.0155 (7)	
O3	0.3320 (4)	0.6807 (3)	0.8313 (2)	0.0146 (7)	
O4	0.6627 (4)	0.6759 (3)	0.7968 (3)	0.0187 (8)	
O5	0.4982 (6)	0.8616 (3)	0.7455 (3)	0.0273 (9)	
O6	0.7604 (5)	0.4761 (3)	0.6609 (3)	0.0313 (10)	
O7	0.2779 (6)	0.7443 (3)	0.6214 (3)	0.0305 (10)	
O8	0.6055 (6)	0.7332 (3)	0.5839 (3)	0.0330 (10)	
O9	0.2086 (5)	0.4954 (3)	0.9257 (3)	0.0200 (8)	
O10	0.9963 (4)	0.6679 (2)	0.8464 (2)	0.0136 (6)	
O11	0.8536 (4)	0.3194 (2)	0.5245 (2)	0.0099 (6)	
O12	0.1790 (4)	0.3351 (2)	0.5557 (2)	0.0100 (6)	
O13	0.5239 (4)	0.3741 (2)	0.5374 (2)	0.0103 (6)	
O14	0.0441 (5)	0.3976 (4)	0.7460 (3)	0.0309 (10)	
O15	0.0494 (5)	0.5469 (3)	0.5840 (4)	0.0347 (10)	

### Atomic displacement parameters (Å^2^)

**Table:**

	*U*^11^	*U*^22^	*U*^33^	*U*^12^	*U*^13^	*U*^23^
Mo1	0.00734 (18)	0.01387 (16)	0.01180 (17)	−0.00106 (15)	0.00059 (14)	−0.00650 (13)
Mo2	0.00731 (17)	0.01417 (17)	0.00660 (17)	−0.00085 (15)	−0.00026 (14)	−0.00219 (13)
Mo3	0.00694 (17)	0.01092 (16)	0.00703 (16)	0.00121 (14)	0.00010 (13)	−0.00095 (13)
Mo4	0.0238 (2)	0.00954 (16)	0.00534 (16)	0.00046 (15)	−0.00095 (16)	−0.00032 (12)
As1	0.0092 (2)	0.01012 (18)	0.00711 (19)	0.00226 (17)	−0.00031 (17)	−0.00295 (15)
K1	0.0510 (9)	0.0557 (8)	0.0355 (7)	0.0067 (8)	0.0015 (8)	−0.0129 (7)
O1	0.032 (2)	0.0155 (16)	0.0235 (19)	−0.0039 (15)	0.0019 (17)	−0.0053 (13)
O2	0.0163 (16)	0.0096 (13)	0.0206 (16)	0.0036 (13)	0.0004 (15)	−0.0043 (12)
O3	0.0107 (16)	0.0233 (15)	0.0098 (14)	−0.0029 (13)	0.0028 (13)	−0.0026 (12)
O4	0.0123 (18)	0.0337 (19)	0.0100 (15)	0.0033 (15)	−0.0020 (13)	−0.0028 (13)
O5	0.054 (3)	0.0143 (14)	0.0135 (15)	−0.006 (2)	0.0064 (17)	−0.0017 (12)
O6	0.0116 (18)	0.044 (2)	0.038 (2)	0.0011 (17)	0.0001 (17)	−0.0319 (18)
O7	0.039 (3)	0.0190 (16)	0.033 (2)	0.0086 (17)	−0.020 (2)	0.0029 (16)
O8	0.050 (3)	0.029 (2)	0.0200 (18)	−0.0101 (19)	0.015 (2)	0.0005 (15)
O9	0.0235 (19)	0.0150 (15)	0.0214 (18)	0.0024 (15)	−0.0081 (17)	−0.0046 (13)
O10	0.0103 (15)	0.0262 (15)	0.0042 (13)	0.0015 (14)	−0.0028 (13)	−0.0014 (11)
O11	0.0085 (15)	0.0130 (14)	0.0082 (14)	−0.0021 (12)	−0.0028 (12)	−0.0010 (11)
O12	0.0074 (15)	0.0102 (13)	0.0123 (14)	0.0006 (11)	0.0012 (12)	−0.0019 (11)
O13	0.0084 (15)	0.0158 (13)	0.0066 (13)	−0.0002 (12)	−0.0003 (12)	−0.0050 (11)
O14	0.024 (2)	0.053 (2)	0.0156 (16)	0.0141 (19)	−0.0036 (16)	−0.0148 (16)
O15	0.028 (2)	0.0197 (17)	0.057 (3)	−0.0052 (17)	0.021 (2)	−0.0103 (17)

### Geometric parameters (Å, º)

**Table:**

Mo1—O14^i^	1.674 (4)	Mo4—O8	1.703 (4)
Mo1—O15^i^	1.682 (4)	Mo4—O5	1.947 (3)
Mo1—O12^i^	1.977 (3)	Mo4—O2	1.964 (3)
Mo1—O11	2.005 (3)	Mo4—O3	2.213 (3)
Mo1—O6	2.048 (4)	Mo4—O4	2.237 (3)
Mo2—O1	1.675 (4)	As1—O6	1.656 (4)
Mo2—O4	1.735 (3)	As1—O2	1.661 (3)
Mo2—O10	1.890 (4)	As1—O5^vi^	1.662 (3)
Mo2—O11^ii^	1.936 (3)	As1—O13	1.685 (3)
Mo2—O13^ii^	2.289 (3)	K1—O14^iii^	2.558 (4)
Mo2—O12^iii^	2.388 (3)	K1—O15^i^	2.662 (4)
Mo3—O9	1.677 (3)	K1—O8	2.676 (5)
Mo3—O3	1.742 (3)	K1—O8^vii^	2.745 (5)
Mo3—O12^iv^	1.934 (3)	K1—O7^i^	2.771 (5)
Mo3—O10^v^	1.946 (3)	K1—O9^iii^	3.106 (4)
Mo3—O13^iv^	2.237 (3)	K1—O7^vii^	3.118 (5)
Mo3—O11^iii^	2.300 (3)	K1—O10	3.290 (3)
Mo4—O7	1.693 (4)	K1—O4	3.423 (4)
			
O14^i^—Mo1—O15^i^	108.2 (2)	O9—Mo3—O13^iv^	90.84 (15)
O14^i^—Mo1—O12^i^	97.31 (16)	O3—Mo3—O13^iv^	162.09 (13)
O15^i^—Mo1—O12^i^	97.29 (16)	O12^iv^—Mo3—O13^iv^	84.88 (12)
O14^i^—Mo1—O11	129.64 (18)	O10^v^—Mo3—O13^iv^	72.32 (12)
O15^i^—Mo1—O11	121.71 (18)	O9—Mo3—O11^iii^	165.14 (15)
O12^i^—Mo1—O11	83.60 (13)	O3—Mo3—O11^iii^	89.84 (13)
O14^i^—Mo1—O6	92.10 (18)	O12^iv^—Mo3—O11^iii^	72.79 (11)
O15^i^—Mo1—O6	90.35 (19)	O10^v^—Mo3—O11^iii^	79.19 (12)
O12^i^—Mo1—O6	165.36 (13)	O13^iv^—Mo3—O11^iii^	76.06 (11)
O11—Mo1—O6	81.76 (14)	O7—Mo4—O8	104.3 (2)
O1—Mo2—O4	103.02 (19)	O7—Mo4—O5	97.95 (19)
O1—Mo2—O10	105.44 (17)	O8—Mo4—O5	95.13 (18)
O4—Mo2—O10	99.00 (15)	O7—Mo4—O2	94.84 (16)
O1—Mo2—O11^ii^	99.25 (16)	O8—Mo4—O2	95.74 (17)
O4—Mo2—O11^ii^	103.76 (14)	O5—Mo4—O2	160.62 (14)
O10—Mo2—O11^ii^	141.44 (13)	O7—Mo4—O3	90.67 (18)
O1—Mo2—O13^ii^	87.18 (16)	O8—Mo4—O3	165.03 (19)
O4—Mo2—O13^ii^	168.11 (14)	O5—Mo4—O3	82.88 (13)
O10—Mo2—O13^ii^	72.05 (12)	O2—Mo4—O3	82.47 (13)
O11^ii^—Mo2—O13^ii^	80.21 (12)	O7—Mo4—O4	165.11 (18)
O1—Mo2—O12^iii^	165.31 (15)	O8—Mo4—O4	90.47 (19)
O4—Mo2—O12^iii^	90.04 (14)	O5—Mo4—O4	78.55 (16)
O10—Mo2—O12^iii^	78.65 (12)	O2—Mo4—O4	85.37 (14)
O11^ii^—Mo2—O12^iii^	70.72 (12)	O3—Mo4—O4	74.58 (12)
O13^ii^—Mo2—O12^iii^	80.64 (10)	O6—As1—O2	110.01 (18)
O9—Mo3—O3	104.30 (17)	O6—As1—O5^vi^	112.4 (2)
O9—Mo3—O12^iv^	99.44 (15)	O2—As1—O5^vi^	102.26 (18)
O3—Mo3—O12^iv^	101.66 (14)	O6—As1—O13	110.60 (17)
O9—Mo3—O10^v^	103.77 (16)	O2—As1—O13	110.10 (15)
O3—Mo3—O10^v^	94.47 (15)	O5^vi^—As1—O13	111.22 (16)
O12^iv^—Mo3—O10^v^	147.40 (12)		

Symmetry codes: (i) *x*+1, *y*, *z*; (ii) −*x*+3/2, −*y*+1, *z*+1/2; (iii) −*x*+1, *y*+1/2, −*z*+3/2; (iv) −*x*+1/2, −*y*+1, *z*+1/2; (v) *x*−1, *y*, *z*; (vi) −*x*+1, *y*−1/2, −*z*+3/2; (vii) *x*+1/2, −*y*+3/2, −*z*+1.
